# Protective Ability of Biogenic Antimicrobial Peptide Microcin J25 Against Enterotoxigenic *Escherichia Coli*-Induced Intestinal Epithelial Dysfunction and Inflammatory Responses IPEC-J2 Cells

**DOI:** 10.3389/fcimb.2018.00242

**Published:** 2018-07-13

**Authors:** Haitao Yu, Xiuliang Ding, Lijun Shang, Xiangfang Zeng, Hongbin Liu, Ning Li, Shuo Huang, Yuming Wang, Gang Wang, Shuang Cai, Meixia Chen, Crystal L. Levesque, Lee J. Johnston, Shiyan Qiao

**Affiliations:** ^1^State Key Laboratory of Animal Nutrition, China Agricultural University, Beijing, China; ^2^Beijing Key Laboratory of Bio-feed Additives, China Agricultural University, Beijing, China; ^3^National Feed Engineering Technology Research Center, Beijing, China; ^4^Department of Animal Sciences, South Dakota State University, Brookings, SD, United States; ^5^Swine Nutrition and Production, West Central Research and Outreach Center, University of Minnesota, Morris, MN, United States

**Keywords:** enterotoxigenic *Escherichia coli* K88, antibacterial activity, inflammatory responses, biogenic antimicrobial peptide Microcin J25, intestinal epithelial barrier, tight junctions, intestinal porcine cells line

## Abstract

Poison of intestinal induce severe health problems in human infants and young animals due to contaminating foods and feedstuffs. With the emergence of public health concerns and high-speed diffuse of drug-opposition of bacteria, the adoption of antimicrobial peptides as potential candidates in treating pathogen infections raised up. Nature Microcin J25 (MccJ25), a class of lasso peptides separated from a fecal strain of *E. coli*, has been replied to display powerful antimicrobial behavior. Herein, the study was to assess the usefulness of biogenic MccJ25 in the prophylaxis of ETEC K88 infection in IPEC-J2 cells. *In vitro* antimicrobial activity against ETEC K88 and cytotoxicity of biogenic MccJ25 were determined first. To further understand how biogenic MccJ25 mediates its impact, ETEC K88 adhesion in cells, membrane permeability [as indicated by reduced release of lactate dehydrogenase (LDH)], transepithelial electrical resistance (TEER), barrier function, and proinflammatory cytokines levels were determined in IPEC-J2 cells after treatment with biogenic MccJ25 and challenge with ETEC K88. Biogenic MccJ25 had a minimum inhibitory concentration of 0.25 μg/mL against ETEC K88, decreased ETEC K88 adhesion in cells and did not cause cytotoxicity toward cells. Furthermore, biogenic MccJ25 protects against ETEC-induced barrier dysfunction by increasing the TEER, decreasing the LDH and promoting tight junction proteins (TJPs) by promoting the assembly of occludin and claudin-1 in the tight junction complex. Biogenic MccJ25 was further found to relieve inflammation responses through modulation of interleukine-6, IL-8 and tumor necrosis factor-α levels via inhibition of mitogen-activated protein kinase (MAPK) and nuclear factor κB activation. In summary, biogenic MccJ25 can protects against ETEC K88-induced intestinal damage and inflammatory response, recommend the hidden adoption of biogenic MccJ25 as a novel prophylactic agent to reduce pathogen infection in animals, food or humans.

## Introduction

The intestinal upper protective screen is the primary platoon of ward against the incursion of cause of disease microorganisms or poison components (Farhadi et al., 2003). The increase in pathogen bacteria which adhesion in intestinal epithelial cells influence intestinal function of permeable changes the structure of gallbladder microbiota, also causes inflammation (Yuhan et al., 1997; Ahmed et al., 2016; Landy et al., 2016). In particular, impaired upper protective screen effect cause damage to immune homeostasis, and increases flame in the bowel. Disruption of gut micro-ecology and barrier function is related to many intestinal illness, including phlogistic intestine illness and intestinal cause of disease infection, and autism spectrum disorders, Parkinson's disease and anxiety disorder (Lu et al., 2014; Fuente-Nunez et al., 2018).

There is increasing evidence that human infants and young animals experience a high rate of intestinal diseases caused by enterotoxigenic *Escherichia coli* (ETEC) acquired by ingestion of contaminated food or water (Black, 1990). This well-known ETEC can destroy epithelial barrier, increase intestinal permeability, cause inflammatory responses to human or mammals, which intensifies the systemic inflammation and finally damages intestinal health (Johnson et al., 2010; Wu et al., 2016; Brown et al., 2018). Intestinal epithelial cells can also maintain immune homeostasis through interacting with commensal bacteria. The gut microbiota has significant effects on animal or human health, when the intestinal epithelial barrier is damaged, the gut microbiome will have a risk of inflammatory responses and infection (Hooper and Gordon, 2001; Park et al., 2010; Chen et al., 2015; Fuente-Nunez et al., 2018). Due to the important statue of crosstalk among gut microbiota, intestinal barrier, and inflammatory responses in gut micro-ecology (Chen et al., 2015; Zhang et al., 2017; Fuente-Nunez et al., 2018), it is critical to understand the antibacterial effect on pathogens to fullfil the full potential and consequences of antimicrobial agents.

Generally, antibiotics are usually regarded as the most effective intervention in human and veterinary medicine. However, the appearing of antibiotic counteractive bacteria has effected in an enhance in curing miscarriage proportion for infectious illness causing a global public health disturbance (Boucher et al., 2009). In addition, in recent years, in despite of the remarkable increase in an attempt to find new medicine, no new antibiotics have been approved for clinical use, particularly those to treat Gram-negative enteric bacteria (Boucher et al., 2009; Rabanal et al., 2015). Therefore, the emergence of antibiotic-counteractive microorganism has stressed a demand of find another to cure human and mammal infection.

Exploiting antimicrobial peptides (AMPs) as an antibiotic-single way to heighten the hosts' resistance and dominate infections is of particular concern. The application of AMPs as a potential intervention has attracted widespread attention to restrict the use of antibiotics in food and animal operation (Wu et al., 2012; Wang et al., 2014, 2017; Yu et al., 2017). Nature Microcin J25, a plasmid-encoded, small antimicrobial peptide synthesized in the ribosome, is a well-studied member of the class of lasso peptides (Salomón and Farias, 1992). Due to the high stability of the fascinating lasso structure and strong antibacterial activity, biogenic MccJ25 has attracted considerable interest for further applications (Blond et al., 1999; Sable et al., 2000).

In our laboratory, we design a biogenic MccJ25 high-efficiency expression vector using standard recombinant DNA (Figure S1). The biogenic MccJ25 has been shown to have pleiotropic functions not only to eradicate pathogens in the gastrointestinal tract but also to maintain homeostasis, as well as decrease in intestinal permeability and stimulates an anti-inflammatory response (Yu et al., 2017). Consequently, biogenic MccJ25 can be favorable to the whole gallbladder health and offer convervation against pathogen infections. Comparing to some other synthesis or recombinant expression antimicrobial peptides (Herbel et al., 2015; Zong et al., 2016; Cao et al., 2018), the *in vitro* efficacy against ETEC K88 and cytotoxicity of biogenic MccJ25 have not been investigated. Here, we evaluated the *in vitro* antibacterial activity of biogenic MccJ25 against ETEC K88 and investigated the protective capacity of biogenic MccJ25 against ETEC K88-induced the intestinal protective screen disfunction and inflammatory responses model.

## Materials and methods

### Biogenic MccJ25 and bacterial strain

Comparing chemical composite, recombinant expression was always used to produce AMPs in many laboratories (Chen et al., 2009; Herbel et al., 2015; Cao et al., 2018). *Escherichia coli* is the mass home and abroad used master bacteria for the expression of AMPs (Pan, 2012; Herbel et al., 2015), thus in our laboratory, biogenic MccJ25 was generated using a highly efficient expression vector as depicted before with secondary mitigation (Yu et al., 2017). Briefly, pMJ25 expression vector was engineered via an efficient recombinant DNA technology (Figure S1). In particular, the codon-optimized genes coding was ligated construct utterance vector pMJ25. Then pMJ25 was altered into *E. coli* B21. The recombinant bacteria were incubated in sucrose-compound medium at 37°C with 100 μg/L ampicillin in 10L fermenter for 22 h. After hatch, cell supernatant was cropped by centrifuge. The purity of the biogenic MccJ25 was above 99.95% and determined by high-performance juice color-process (HPLC). The approach of purity of biogenic MccJ25 was provided in Supplementary section. The amine sour array of the peptide was GGAGHVPEYFVGIGTPISFYG determined by automatize Edman degradation (model 494 Procise Protein/Peptide Sequencer; Applied Biosystems, Foster City, CA) and a mass spectrometer. The biogenic MccJ25 was emerged as lyophilized dust and deposited at −20°C.

The ETEC K88 (serotype O149:K91, K88ac) strain, also named ETEC F4, conveying F4ac fimbriae is deemed the major pathogen be related with human infants and neonatal diarrhea (Black, 1990; Osek, 2000). The ETEC K88 strain was gained from China Institute of Veterinary Drug Control (Beijing, China) and increased in Mueller-Hinton (MH) broth (Difco, England) on a rotary shaker at 180 rpm for 6 h until reaching the mid-logarithmic phase of growth. Then the cultures were centrifuged at 8500 rpm for 5 min, washed, and re-suspended in frosty PBS to obtain a final bacterial density of 2 × 10^9^ colony forming units (CFU)/mL. The ETEC K88 was the test living creature used from beginning to end this study.

### Minimum inhibitory concentration

The minimal inhibitory concentration (MIC) of the biogenic MccJ25 was determined in sterilized 96-well microplates (Costar, Corning Inc., Corning, NY, USA) using microdilution assays as described previously (Wang et al., 2017). Briefly, biogenic MccJ25 was dismissed in distilled water, and serial 2-fold dilutions were made in MH broth using 96-well microplates. Finally, the concentration of biogenic MccJ25 ranged from 0.125 to 256 μg/mL. Ten mL of bacteria stay overnight culture mixture was inoculated into each well at a concentration of 1.0 × 10^6^ CFU/mL. The micro plate was hatched at 37°C for 24 h and bacterial growth was surveyed by an alter in absorbance at 600 nm using a microplate auto reader (Bio-Rad Laboratories, Hercules, CA). Positive (media with inoculum) and passive controls (media only) were included. The MIC was determined as the lowest concentration of biogenic MccJ25 that restrained bacteria growth (be short of raise in absorbance reading). The dissect were performed in triplicate.

### Time-killing assay

The ETEC K88 (1.0 × 10^6^ CFU/mL) was evolved in MH broth including a range of density of biogenic MccJ25 (0, 0.25, and 0.5 × MIC). All creatures were hatched at 37°C in a 180 rpm shaker bath. Sampling times included 0, 10, 20, 30, 40, 50, 60, 70, 80, 90, 100, 110, and 120 min. Then, time-kill curves of biogenic MccJ25 were determined as before depicted (Wang et al., 2017) and the killing rate was determined by plotting the log CFU/mL against time.

### Antibacterial activity

As mentioned before (Wang et al., 2017), the method of determining biogenic MccJ25 antibacterial activity is Agarose Diffusion method. ETEC K88 was inoculated into the MH medium with a content of 1% agar, and the duration of the culture was a whole day. The concentration of bacteria obtained by culture was close to 10^6^ CFU/ml, and then pour it into the culture dish. The agar medium was first solidified, and then the aseptic cork drill was used to drill it. Finally, the biogenic MccJ25 powder was put into the sterilized water and fully stirred to make the biogenic MccJ25 evenly distributed in the sterilized water. At this time, its concentration was about 0.25 μg/mL. Take out the sample of 200 μL and introduce it into the 8-mm wells. After that, wells were incubated in room temperature for 1 h and then put in a temperature of about 37°C for 24 h. A negative control using water was also included.

### Cells culture

The IPEC-J2 cells were kindly provided by Dr. Guoyao Wu (College Station, Texas A&M University, US). Cells were cultured in Dulbecco's Modified Eagle medium/Nutrient Mixture F-12, 1:1 mixture of DMEM and Ham's F-12 (DMEM/Ham's F12 1:1) (Gibco, Merelbeke, Belgium) supplemented 5% (vol/vol) fetal bovine serum (FBS, Gibco, Carlsbad, CA), 1% streptomycin (10,000 g/mL)/penicillin (10,000 U/mL) (Gibco), 5 μg/L ITS (Sciencell, Carlsbad, CA, USA) and 5 μg/L epidermal growth factor (Sciencell, Carlsbad, CA, USA) and maintained under a 95% humidified atmosphere of 5% CO_2_ at 37°C. Cells were maintained with media replenished every day.

### Cytotoxicity studies

To assess whether biogenic MccJ25 affects cell viability, IPEC-J2 cells were seeded on 96-well cell culture plates (Costar, Corning Inc., Corning, NY, USA) at a density of 2 × 10^6^ cells/mL per well. The cell culture plates were incubated at 37°C, 5% CO_2_ for 24 h until 80% confluence was reached. The cells were then treated with the indicated concentration (2–256 μg/mL) of biogenic MccJ25 solution containing DMEM/F12 (1:1) medium for 24 or 48 h. Wells containing untreated cells served as a control. Then, cell viability was determined by the cell counting kit (CCK-8) as previously described (Wang et al., 2017) and the data was expressed as a percentage of control cells.

To identify whether biogenic MccJ25 caused cell membrane damage, lactate dehydrogenase (LDH) in IPEC-J2 cell culture medium was measured using the LDH release assay (Promega, Wisconsin, USA). DMEM/F12 medium (2 × 10^6^ cells/well) containing IPEC-J2 cells was inoculated on a 96-well assay plate (Costar, Corning Inc., Corning, NY, USA) and cultured at 37°C, 5% CO_2_, 24 or 48 h until reaching 80% confluence. Subsequently, biogenic MccJ25 with a concentration range between 2 and 256 μg/mL was added to each well. The cells were incubated at 37°C, 5% CO_2_ for 24 or 48 h. After this time, the medium was collected and measured for LDH activity using the CytoTox 96® Reagent and LDH Detection Kit (Promega, USA). Each group consists of 6 replicates (wells) and the data is expressed as a percentage of control cells.

### Differentiation of intestinal epithelial cells

IPEC-J2 cells (2 × 10^5^) were transformed into a 6-well transwell collagen-coated PTFE filter (pore size 0.4 μm; 4.7 cm^2^; Costar, Corning Inc., Corning, NY, USA) according to the standard protocol. The bottom side of each well and 2.6 mL were on the basolateral side. IPEC-J2 cells were incubated in culture medium for 24 h and freshly re-fed daily before confluence. After that, the cells were fed with a medium containing no FBS to differentiate the cells. To monitor differentiation, transepithelial electrical resistance (TEER) was measured every other day using the Millicell resistance system (Millipore, Darmstadt, Germany) until it usually reached about 2,000 cm^2^.

### Bacteria adhesion assay

The IPEC-J2 cells were seeded onto a 6-well transwell collagen-coated PTFE filter (pore size 0.4 μm; 4.7 cm^2^ Costar, Corning Inc., Corning, NY, USA) and increased to confluence. Before infection, cells were incubated with medium alone or with medium containing MccJ25 (2 μg/mL) for 1 h. The ETEC K88 (10^7^ CFU/mL) was grown to mid-logarithmic photo, sacked and resuspended in central, then increased to the IPEC-J2 cells for 3 h. We select the bacterial concentration and occasion of incubation based on prime tests to allow for bacterial adhesion and film harm without interruption of the cell monolayers. Then, bacteria adhesion was evaluated as before depicted (Xia et al., 2015). Briefly, after hatch, cells were washed five times with PBS to clear other bacteria. Then the cells were bushed five times with PBS, and the cells were conquered to a Triton X-100 condition, following a 5-min incubation and a serious of addition of 800 μL PBS. Ten μL simples of serial 10-fold thinning of cell lysates were placed on Bismuth Sulfite Agar to quantify bacteria. The number of CFU was concluded after all-night trained at 37°C.

### *In vitro* ETEC K88 challenge IPEC-J2 cells experiments

The IPEC-J2 Cells (2 × 10^5^) were planted onto a 6-well transwell fiber-lidded PTFE lauter (pore size 0.4 μm; 4.7 cm^2^; Costar, Corning Inc., Corning, NY, USA) and grown to confluence. After simples were distinguished, cells were treated. The cells were separated into the following four groups: control (untreated), ETEC K88 (treated with a final concentration 4.2 × 10^6^ ETEC K88 for 3 h), biogenic MccJ25 (treated with 2 μg/mL biogenic MccJ25 for 24 h), and biogenic MccJ25 + ETEC K88 (treated with 2 μg/mL biogenic MccJ25 for 24 h before layout to a final concentration 4.2 × 10^6^ ETEC K88 for 3 h). Thereafter, LDH and TEER were determined. In addition, cells and media were collected and stored at −80°C until analyzed. The concentration and mRNA expression of inflammatory, and TJPs abundance were examined.

### Determination of TEER and LDH

The IPEC-J2 Cells (2 × 10^5^) were planted onto a 6-well transwell fiber-lidded PTFE filter (pore size 0.4 μm; 4.7 cm^2^; Costar, Corning Inc., Corning, NY, USA) and grown to confluence. After simples were distinguished, cells were treated. The cells were separated into the following four groups: control (untreated), ETEC K88 (treated with a final concentration 4.2 × 10^6^ ETEC K88 for 3 h), biogenic MccJ25 (treated with 2 μg/mL biogenic MccJ25 for 24 h), and biogenic MccJ25 + ETEC K88 (treated with 2 μg/mL biogenic MccJ25 for 24 h before layout to a final concentration 4.2 × 10^6^ ETEC K88 for 3 h).

To study membrane damage induced by ETEC K88 and protective effect of biogenic MccJ25, the differentiated IPEC-J2 cells were adopted with or without biogenic MccJ25 as indexed for 24 h in the existence or shortage of ETEC K88 for 3 h. LDH activity and TEER were measured, respectively. All data are showed as the significance opposing to those for the control group.

### Determination of proinflammatory cytokines

The levels of cytokines (TNF-α, IL-6, and IL-8) were evaluated after the addition of ETEC K88 to IPEC-J2 cells in the presence or absence of biogenic MccJ25 in DMEM/F12 supplemented with serum (no antibiotics). Cytokine secretion was measured in the culture supernatant using an enzyme-linked immunosorbent assay (ELISA) kit purchased from Liangsichangyuan Bioengineering Institute (Beijing, Chian). The concentration was quantified by measuring the absorbance at 450 nm on a microplate reader (Bio-Rad Laboratories, Hercules, CA).

### Real-time PCR analysis

Cells were lysed directly in TRIzol (Invitrogen, Carlsbad, CA, USA). Extract total RNA according to the manufacturer's instructions. The first strand cDNA was synthesized by reverse transcription of 1 μg of total RNA using the PrimeScript First Strand cDNA Synthesis Kit (Takara, Dalian, China) according to the manufacturer's protocol and stored at −80°C. Real-time PCR was performed on an Applied Biosystems 7500 real-time PCR system (Applied Biosystems, Singapore) using SYBR Green PCR Master Mix (Takara, Dalian, China) as previously described (Liu et al., 2017). β-actin was used as an endogenous control. The primers used are listed in Table S1. All reactions were performed in triplicate.

### Western blot and immunofluorescence analysis

As described previously (Wang et al., 2017), cells were harvested by Western blot to analyze the abundance of the protein. Membranes were incubated with primary antibodies [occludin, claudin-1, ZO-1, P38, p-P38 and NF-κB (P65, P-p65) (Santa Cruz Biotechnology, USA)] overnight at 4°C and then washed. TBST 3 times in 15 min. The membrane was then incubated with horseradish peroxidase (HRP)-conjugated secondary antibody (Applygen Technology, Inc., Beijing, China) for 1 h at room temperature. Signals were detected using the ImageQuant LAS 4000 mini-system (GE Healthcare Bio-sciences AB, Inc., Sweden) using Western Blot Brightness Reagent (Applygen, Beijing, China) and passed through Image Quant TL Software (GE Healthcare Life Science) for the gel imaging system.

The expression levels of intercellular tight junction proteins (TJPs) occludin, and claudin-1 were evaluated by immunofluorescence microscopy as previously described (Qin et al., 2009; Donato et al., 2010). Briefly, IPEC-J2 cells were incubated with a rabbit anti-occludin Ab and a rabbit anti-claudin-1Ab (Abcam, USA) and then with FITC-conjugated goat anti-rabbit secondary Ab. After washing with PBS, Cells were removed from the plastic support, mounted on glass slides with Vectashield containing DAPI, and examined on a Leica TCS SP5 confocal laser microscope (Keyence, Osaka, Japan).

### Statistical analysis

The results were described as mean ± standard error of the mean (SEM). The data was analyzed by one-way ANOVA using a SAS system (version 9.2, SAS Institute, Inc., Cary, NC). Student-Newman-Keuls multiple comparison test was used to determine the difference between treatments. All data was visualized using GraphPad Prism 6 software (Graphpad Software Inc., San Diego, CA). Statistical significance was expressed using *P* < 0.05.

## Results

### *In vitro* antimicrobial effects of biogenic MccJ25

**Figure 1:**
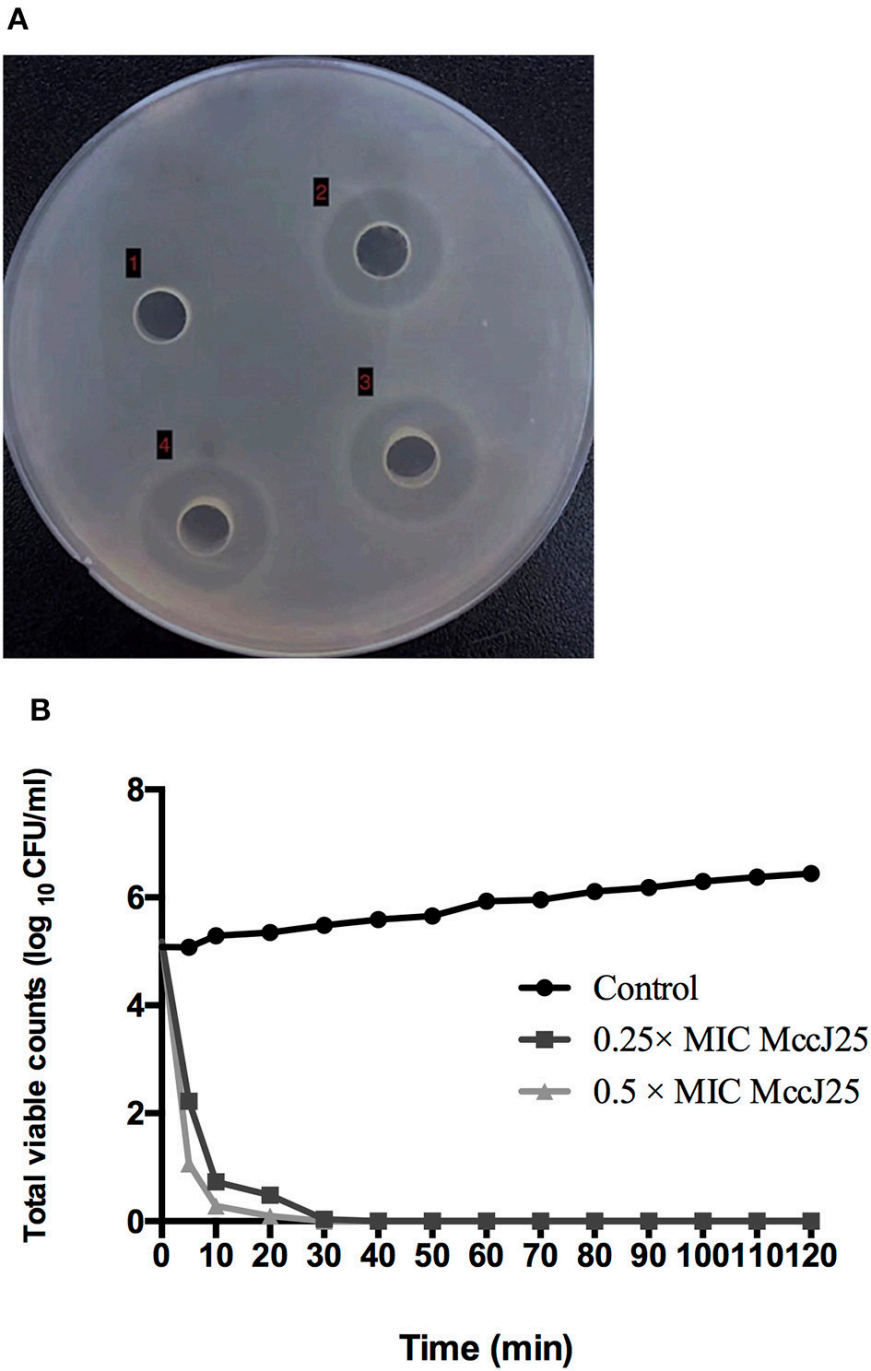
Antimicrobial peptide biogenic MccJ25 kills ETEC K88. **(A)** Photo of inhibition zone assay of ETEC K88 treated with and without biogenic MccJ25 at MIC level. Well (1) represents control with ETEC K88. (2), (3), and (4) were ETEC K88 treated with 0.25 μg/mL biogenic MccJ25. **(B)** Time-kill curves of ETEC K88 incubated in MH broth medium containing different concentration biogenic MccJ25 (0.25-/0.5- fold MIC). Assays were performed in triplicate.

First, the antimicrobial activity of biogenic MccJ25 toward ETEC K88 was evaluated using an MIC assay. Biogenic MccJ25 was highly effective against ETEC K88 at MIC of 0.25 μg/mL. No growth was observed in the wells supplemented with biogenic MccJ25 in the MIC (data not shown). In addition, the antimicrobial effect of biogenic MccJ25 was also evaluated by the agar diffusion method (Figure 1A). When the biogenic MccJ25 at the MIC was transferred to a well prepared on a solidified agarose mixed with ETEC K88, a distinct zone of inhibition was observed.The time-killing kinetic curve showed that an obvious decrease in bacterial growth appeared after 20 and 30 min exposure to biogenic MccJ25, indicating that ETEC K88 was rapidly killed by biogenic MccJ25 within 0.5 h (Figure 1B).

### Cytotoxicity studies

**Figure 2:**
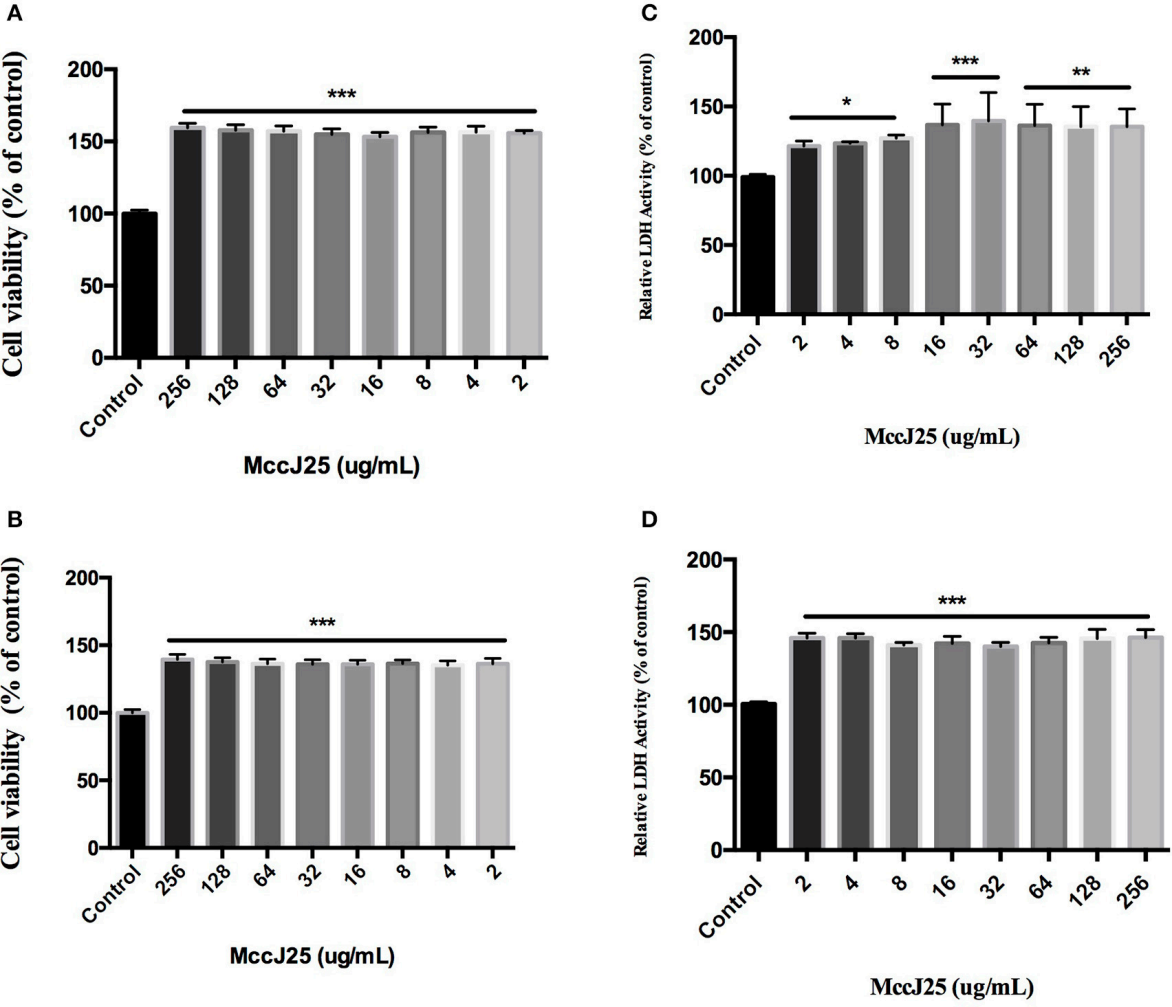
CCK-8 and LDH assay with IPEC-J2 cells shows that antimicrobial peptide biogenic MccJ25 do not induce cytotoxicity. IPEC-J2 cells were cultured with or without biogenic MccJ25 (2–256 μg/mL) for 24 or 48 h. Viability of IEPC-J2 cells after treatment with different McJ25 concentrations for 24 h **(A)** and 48 h **(B)**. LDH activity in IPEC-J2 cell culture medium after treatment with different McJ25 concentrations for 24 h **(C)** and 48 h **(D)**, respectively. Data are means ± SEMs of three independent experiments, *n* = 6. The asterisk denotes a significant difference compared with control group (*P < 0.05, **P < 0.01,***P < 0.001).

Since the basic goal of this study was to develop biogenic MccJ25 as a safe alternative antibacterial agent, the cytotoxicity of biogenic MccJ25 was tested. Cell viability was determined using CCK-8 assay after 24 and 48 h of treatment with different concentrations of biogenic MccJ25. As shown in Figures 2A,B, at 24 and 48 h, various biogenic MccJ25 concentrations increased cell viability compared to the control group (*P* < 0.001). Even at a concentration of 256 μg/mL, no significant effect of cultured cells on the cells was detected.

To further quantify the toxicity of biogenic MccJ25, the LDH assay was conducted. The cells treated with different concentrations of biogenic MccJ25 for 24 and 48 h, did not significantly increase LDH release compared with control group even at a concentration of 256 μg/mL (Figures 2C,D). These results indicated that the treatment with biogenic MccJ25 maintains the integrity of the cellular membrane in IPEC-J2 cells.

### Effects of biogenic MccJ25 on ETEC K88 adhesion in cells

**Figure 3:**
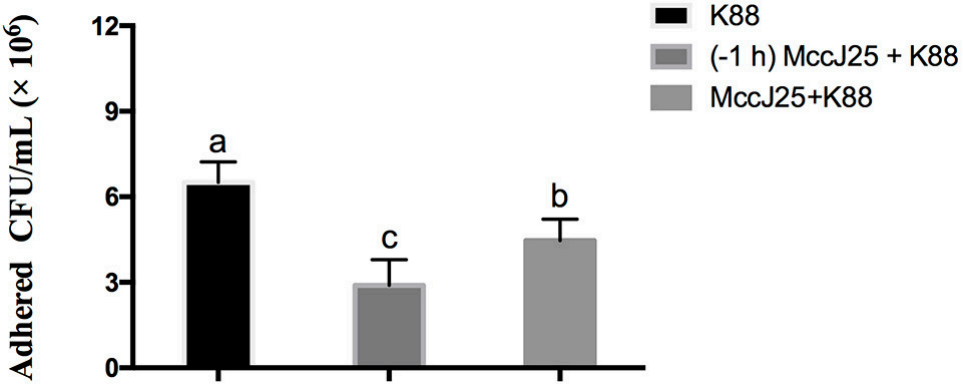
Protective effects of antimicrobial peptide biogenic MccJ25 against ETEC K88 adhesion to IPEC-J2 cell monolayers. IPEC-J2 cells were cultured with or without biogenic MccJ25(2 μg/mL) for1 h in the absence or presence of ETEC K88 for 3 h. The adhesion of ETEC K88 in the IPEC-J2 cells was determined. Data are means ± SEMs of three independent experiments, *n* = 8. Different superscript lowercase letters within each group mean significantly different (*P* < 0.05).

To determine whether biogenic MccJ25 protects cell monolayers from ETEC K88 adhesion in IPEC-J2 cells through its antibacterial activity, *in vitro* bacterial adhesion assay ETEC K88 was performed. This experiment was conducted in two environments. IPEC-J2 cells were treated with biogenic MccJ25 for 1 h and then washed off before addition of bacteria, or kept in culture medium during infection. Compared to the control group, the two washed-out and consistent biogenic MccJ25 groups significantly reduced the ETEC K88 count at 2 μg/mL (*P* < 0.01) (Figure 3). However, consistent biogenic MccJ25 significantly decreased (*P* < 0.01) ETEC K88 adhesion compared to the flushed biogenic MccJ25 treated group.

### Effects of biogenic MccJ25 on ETEC K88-induced cellular damage

**Figure 4:**
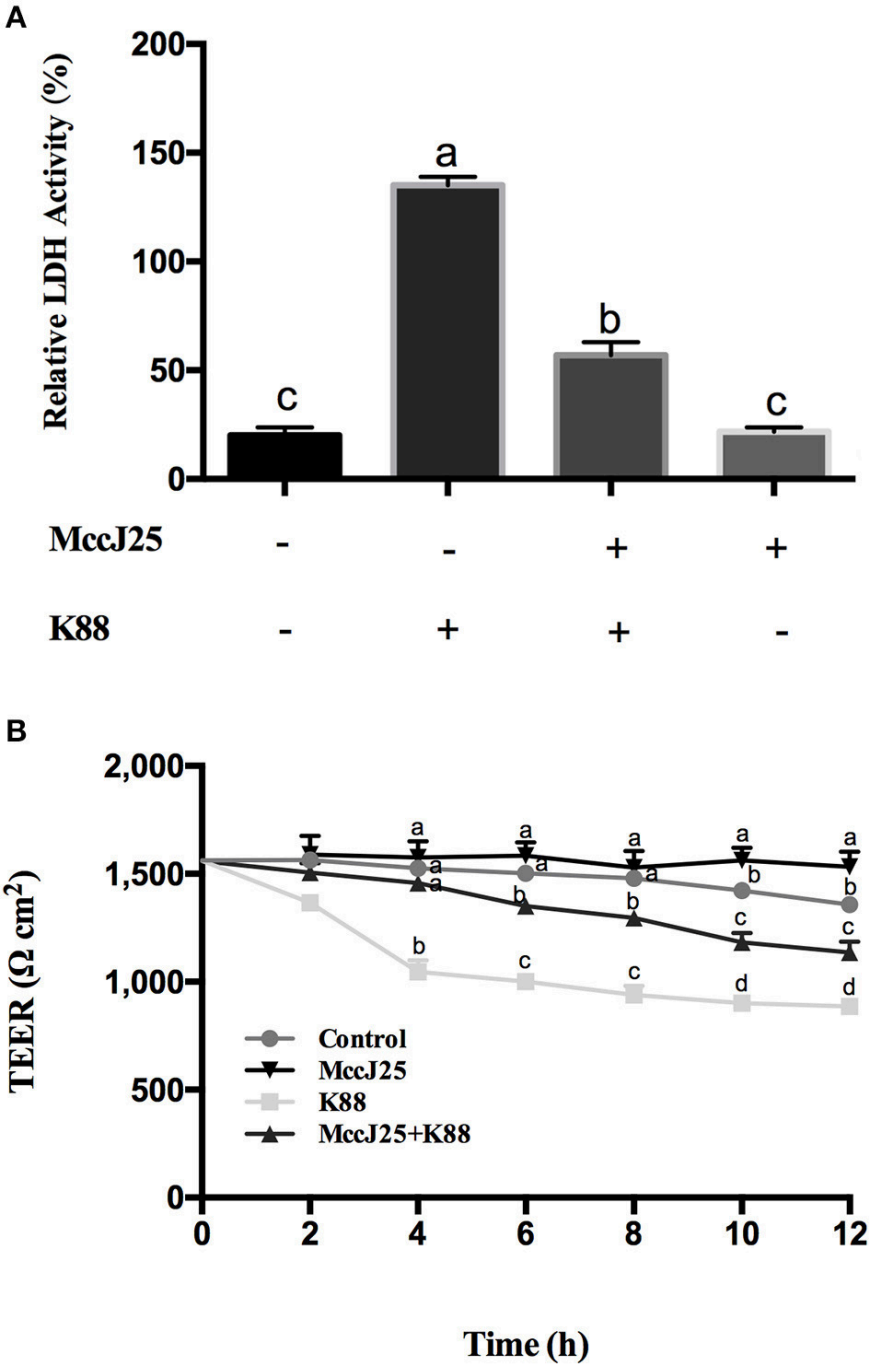
Antimicrobial peptide biogenic MccJ25 inhibited ETEC K88-induced damage to IPEC-J2 cells. IPEC-J2 cells were cultured with or without biogenic MccJ25(2 μg/mL) for 24 h in the absence or presence of ETEC K88 for 3 h. LDH activity **(A)**, TEER **(B)** in the IPEC-J2 cells were determined. Data are means ± SEMs of three independent experiments, *n* = 8. Different superscript lowercase letters within each group mean significantly different (*P* < 0.05).

The intestinal permeability and TEER of biogenic MccJ25 were first tested. As expected, biogenic MccJ25 treatment significantly reduced (*P* < 0.001) LDH release into the medium compared to the control group (Figure 4A). However, the number of LDH in the culture medium of ETEC K88-treated IPEC-J2 cells pretreated with biogenic MccJ25 was significantly reduced compared to the ETEC K88-treated group (*P* < 0.001).

To clarify the role of biogenic MccJ25 on promoting the intestinal junction, the classical TEER method was used to indicate the tight junction integrity. Compared with control group, TEER increased significantly for cells treated with biogenic MccJ25 (*P* < 0.01) at 12 h. The ETEC K88 decrease in induced TEER was attenuated (*P* < 0.01) pretreatment biogenic MccJ25 as compared to that of ETEC K88 treatment group at 12 h (Figure 4B). These findings indicated that biogenic MccJ25 maybe function on the epithelial physical barrier.

### Effects of biogenic MccJ25 on ETEC K88-induced disruption of tight junction gene expression

The intestinal barrier is mainly formed by tight connections. To further investigate the protective effect of biogenic MccJ25 on ETEC K88-induced TJP disruption, such as Claudin-1, occludin, and ZO-1 expression, it was determined by Real-Time PCR and Western blotting.

**Figure 5:**
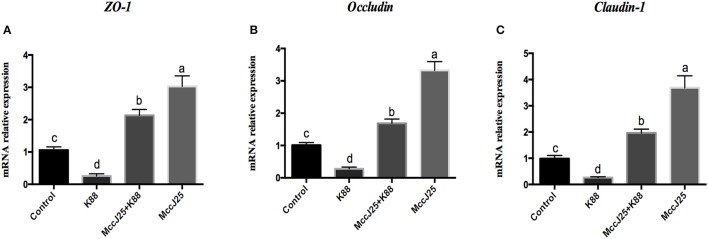
Lasso peptide biogenic MccJ25 increased the mRNA expression of the tight junction proteins ZO-1 **(A)**, occludin **(B)**, and claudin-1 **(C)** in ETEC K88-challenged IPEC-J2 cells. IPEC-J2 cells were cultured with biogenic MccJ25 (2 μg/mL) or without biogenic MccJ25 for 24 h, and then treated with ETEC K88 for 3 h. Cells were collected and relative mRNA expression was analyzed by Real-Time PCR. Date are means ± SEMs of three independent experiments, *n* = 9. Different superscript lowercase letters within each group mean significantly different (*P* < 0.05).

As expected, ETEC K88 treatment significantly decreased (*P* < 0.05) the mRNA relative abundance of the claudin-1 (Figure 5A), occludin (Figure 5B), and zonula occludens-1(ZO-1, Figure 5C) in the absence of biogenic MccJ25. However, the IPEC-J2 cells pre-cultured with biogenic MccJ25 significantly increased (*P* < 0.001) mRNA expression of claudin-1, occludin, and ZO-1. In addition, compared with control, biogenic MccJ25 treatment significantly increased (*P* < 0.001) the mRNA expression of claudin-1, occludin, and ZO-1.

### Effects of Biogenic MccJ25 on ETEC K88-induced disruption of tight junction proteins expression and distribution

**Figure 6:**
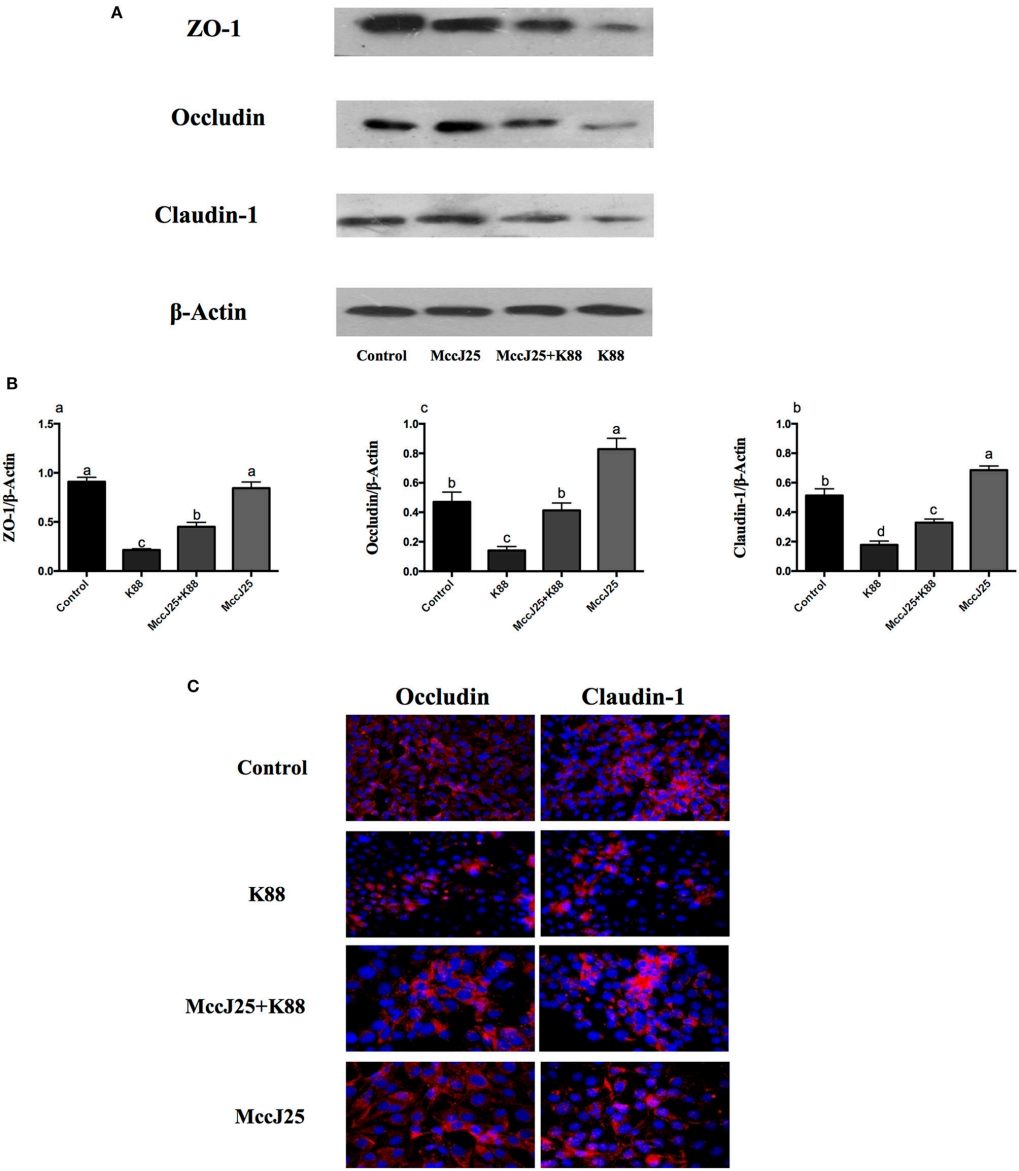
The protective effects of antimicrobial peptide biogenic MccJ25 on intestinal tight junction proteins structure and function in IPEC-J2 cells. IPEC-J2 cells were cultured in the presence or absence of biogenic MccJ25 (2 μg/mL) for 24 h and then treated with ETEC K88 for 3 h. **(A)** Representative panels of ZO-1, Occludin and Claudin proteins in IPEC-J2 cells. **(B)** Cells were collected and protein abundance was determined by Western blot. **(C)** Visualization of the occlaudin and claudin-1 expression (shown in red) in IPEC-J2 cells and its combination with DAPI to visualize the nuclei (shown in blue). Data are means SEMs of three independent experiments, *n* = *3*. Different superscript lowercase letters within each group mean significantly different (*P* < 0.05).

We performed Western blotting experiments to verify the differences in the processing of these TJPs in the expression of protein abundance in ETEC K88 treated IPEC-J2 cells (Figures 6A,B). Compared to all mRNA expression results, cells challenged with ETEC K88 reduced (*P* < 0.001) protein expression of claudin-1, occludin, and ZO-1 compared to all treatment groups (Figures 6A,B). Pretreatment with biogenic MccJ25 significantly inhibited the down-regulated expression of claudin-1, occludin, and ZO-1 induced by ETEC K88 (*P* < 0.05). However, the biogenic MccJ25-treated group did not significantly increase ZO-1 protein expression compared to the control group (Figure 6B).

Differences in claudin-1 and occludin expression levels were further confirmed by confocal immunohistochemistry (Figure 6C), where the expected co-localization of these proteins was confirmed. Consistent with Western blot analysis, biogenic MccJ25 exposure was associated with distribution and irregular cell distribution of claudin-1 and occludin compared to the control group. In addition, pretreatment with biogenic MccJ25 prevented ETEC K88 from disrupting the distribution of claudin-1, occludin, and ZO-1.

### Concentrations and gene expression of proinflammatory cytokines

**Figure 7:**
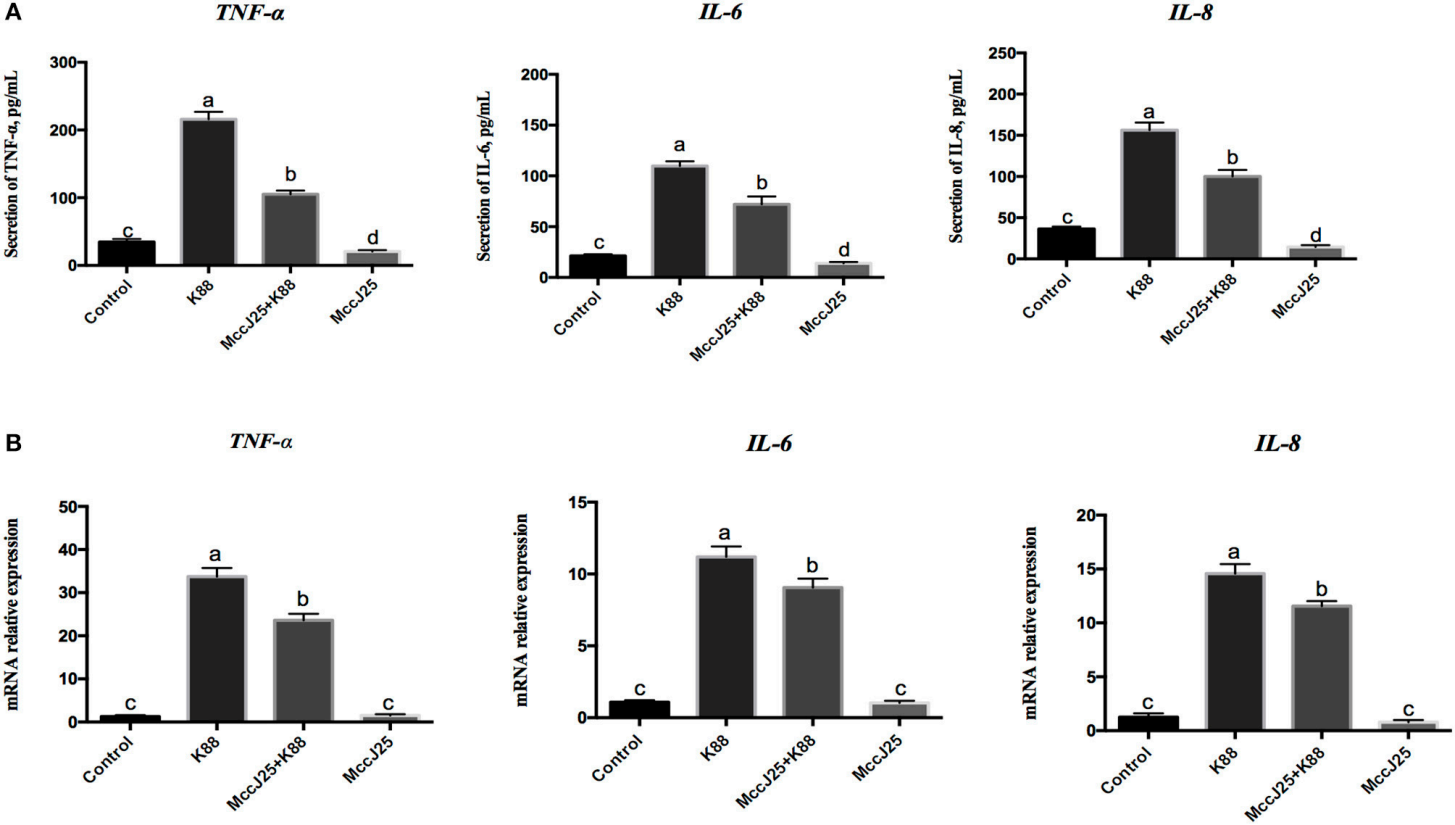
Antimicrobial peptide biogenic MccJ25 decreased proinflammatory cytokines production and mRNA expression in the ETEC K88-challenged IPEC-J2 cells. IPEC-J2 cells were treated with or without biogenic MccJ25 (2 μg/mL) for 24 h and then treated with ETEC K88 for 3 h. Cell supernatant and cells were collected, then proinflammatory cytokines concentrations **(A)** and mRNA expression **(B)** were analyzed. Data are means ± SEMs of three independent experiments, *n* = 9. Different superscript lowercase letters within each group mean significantly different (*P* < 0.05).

The fact that ETEC K88 causes inflammation while biogenic MccJ25 whether has a potent anti-inflammatory effects which prompted us to investigate the role of ETEC K88 on pro-inflammatory cytokines, we analyzed the levels of TNF-α, IL-8, and IL-6 (Figure 7A). Compared with control group, biogenic MccJ25 treatment group significantly decreased (*P* < 0.05) TNF-α, IL-6, and IL-8 secretions. However, there was an increases (*P* < 0.001) in TNF-α, IL-6, and IL-8 levels in IPEC-J2 cells after treatment with ETEC K88 for 3 h compared with all treatment groups (Figure 7A). Pre-treatment with biogenic MccJ25 before exposure to ETEC K88 significantly reduced the levels of IL-6 (*P* < 0.05), IL-8 (*P* < 0.001), and TNF-α (*P* < 0.001).

In an agreement with the proinflammatory cytokines secretion results, when ETEC K88 challenged there was an increase (*P* < 0.001) in TNF-α, IL-6, and IL-8 gene expression in IPEC-J2 cell culture supernatants compared with control group (Figure 7B), whereas ETEC K88 increased the TNF-α, IL-6, and IL-8 gene expression appeared to be prevented by biogenic MccJ25. Compared with control group, biogenic MccJ25 treatment group did not significant decreased TNF-α, IL-6, and IL-8 expression.

### Effects of biogenic MccJ25 on mitogen-activated protein kinase and nuclear factor κB pathways in IPEC-J2 cells

**Figure 8:**
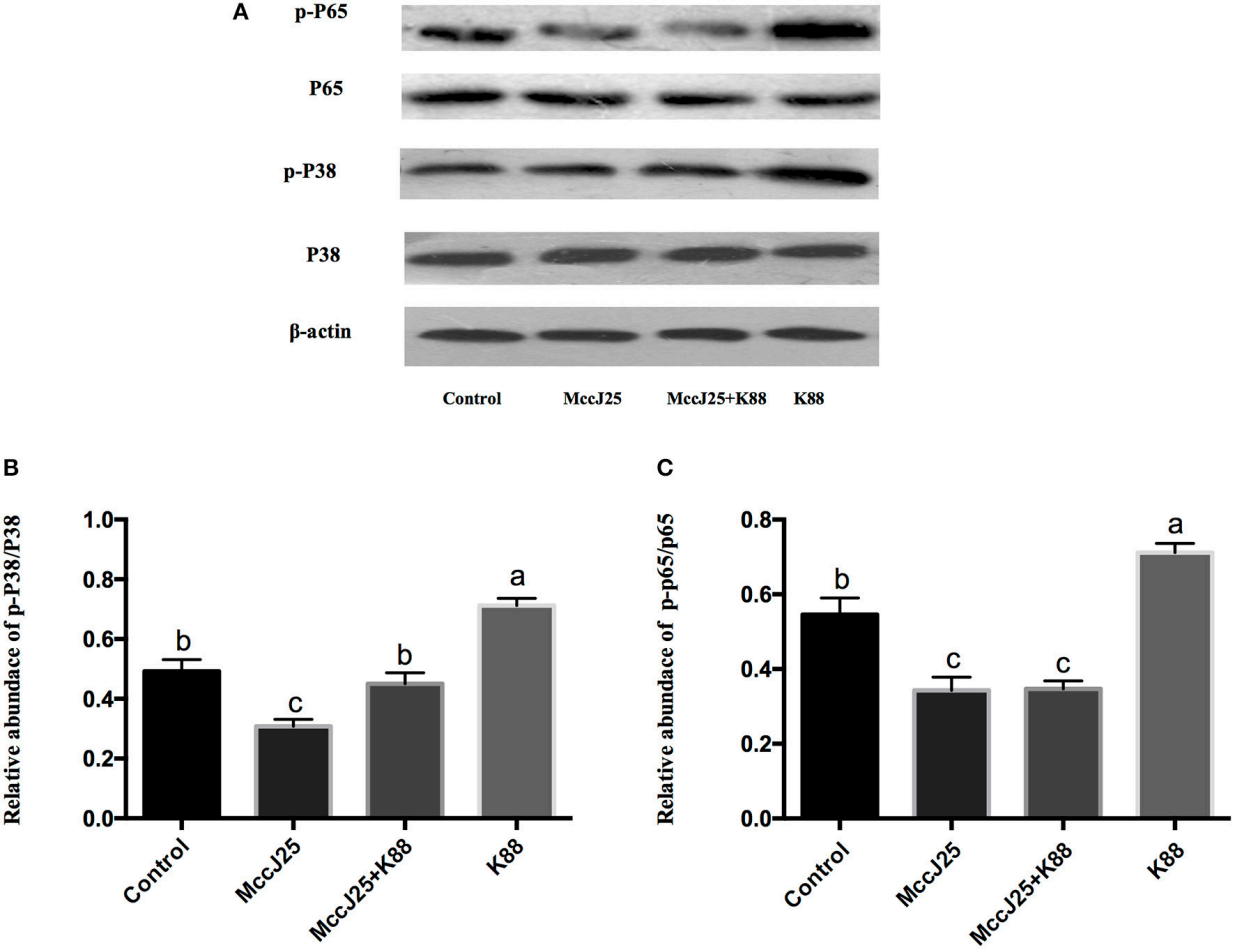
Western blotting analysis of P38 MAPK and NF- κB pathway activation in IPEC-J2 cells. Differentiated monolayers IPEC-J2 cells were pretreated or not with biogenic MccJ25 (2 μg/mL) for 24 h and then treated with ETEC K88 for 3 h. Cells were collected after ETEC K88 challenge. **(A)** Western blotting bands of P38, p-P38 and phosphorylated NF-κB. **(B)** Relative protein abundance of p-P38 and **(C)** phosphorylated NF-κB. Results are given as means ± SEMs of three independent experiments, *n* = 3. Different superscript lowercase letters within each group mean significantly different (*P* < 0.05).

Following the above experiments, biogenic MccJ25 can relieve inflammation responses which prompted us to examined the interaction between biogenic MccJ25 in the NF-κB and mitogen-activated protein kinase (MAPK) pathways, which is a key cellular cascade involved in inflammation. The phosphorylated NF-κB (Figures 8A,C) and P38 (Figures 8A,B) protein abundance was significantly increased (*P* < 0.001) in ETEC K88 group compared with all treatment groups. Compared with the control group, biogenic MccJ25 significantly reduced (*P* < 0.05) the phosphorylated nuclear factor κB (NF-κB) and P38 protein abundance. Additionally, cells pre-treated with biogenic MccJ25 showed significantly decreased (*P* < 0.01) phosphorylated NF-κB protein abundance compared with ETEC treatment group. Importantly, pretreatment with biogenic MccJ25 significantly reduced (*P* < 0.01) the phosphorylated P38 protein abundance compared with ETEC and Control groups.

## Discussion

The present study was to examined the potential protective ability of biogenic MccJ25 against ETEC K88-induced intestinal barrier disfunction in IPEC-J2 cells. Our findings in this study revealed that the biogenic MccJ25 showed no cytotoxicity in the IPEC-J2 cell line based on LDH and CCK-8 assays, while it exerted strong antimicrobial activity on ETEC K88 and significantly reduced ETEC K88 adhesion in cells. In addition, the biogenic MccJ25 was shown to be effective against ETEC K88-induced intestinal barrier dysfunction and to reduce inflammatory responses.

In view of the increase in antibiotic-resistant microorganisms (such as *E. coli, Salmonella, Campylobacter jejuni*, and *Staphylococcus aureus*) and the lack of alternative treatments for infectious diseases (Xiao et al., 2011; Ma et al., 2016; Wang et al., 2017). It is necessary to actively develop alternative methods to control the spread of this pathogen and homologous infections. Nature MccJ25 is a well-studied member of the class of lasso peptides. It was isolated from a fecal strain of *E. coli* AY25 and had strong antibacterial activity (Salomón and Farias, 1992; Blond et al., 1999; Sable et al., 2000). In our laboratory, the biogenic MccJ25 was generated using a highly efficient expression vector as described previously (Yu et al., 2017). Some studies have been showed that recombinant expression is always conducted to produce therapeutic proteins, which are used in many laboratories for large scale expression of AMPs (Chen et al., 2009; Herbel et al., 2015; Cao et al., 2018). A previous study has also been indicated that microcins can conceivably be utilized as a targeted strategy to treat infection disease and enterobacterial colitis during administration to pathogen-infected and inflamed animals (Sassone-Corsi et al., 2016). In summary, there is a need to thoroughly evaluate biogenic MccJ25 to assess its impacts on pathogens. To address this need, in the first phase of the present study, we worked on evaluating the antibacterial activity against ETEC K88 *in vitro*. We found that both the clear zones of inhibition and the low MIC (0.25 μg/mL) demonstrated that biogenic MccJ25 can effectively inhibit the growth of ETEC K88. Time-kill assays also illustrated that biogenic MccJ25 had a powerful killing effect on ETEC K88. Consistent with the previous studies, biogenic MccJ25 had been shown to extremely strong bactericidal activity in the 5–500 nanomolar range against *E. coli, Salmonella*, and *Shigella* strains (Blond et al., 1999; Sable et al., 2000). Compared to animal experiments, *in vitro* studies of cell culture are always the first step in studying how drugs respond because they are relatively cost-effective and simple, easy to handle, and ethically unclear (Ma et al., 2017). In this study, we selected highly similar IPEC-J2 cells between pigs and humans, and because it is a better model for normal intestinal epithelial cells to study pathogen-host interaction and gut barrier function (Skjolaas et al., 2006; Schmidt et al., 2008; Wu et al., 2016). In our study, we found that biogenic MccJ25 exert strong antimicrobial activity without raising IPEC-J2 cells cytotoxicity that did not cause cellular membrane damage and reduction in cell activity even at high concentrations. Consistent with the previous studies which found that AMPs sublancin, porcine lactoferrin peptide LF-6 or chitosan microparticles also did not cause cytotoxicity in intestinal cells (Jiang et al., 2016; Ma et al., 2017; Wang et al., 2017). On the basis of the cytotoxicity findings, we choose the lowest doses (2 μg/mL) biogenic MccJ25 to investigate the impacts on following experiments.

Because of the antimicrobial ability of biogenic MccJ25 to against ETEC K88, we investigate the biogenic MccJ25 on the surface of the IPEC-J2 cells inhibits the capacity of potential pathogens. The previous research which suggested that AMP CB-F on the surface of the intestinal epithelial cells inhibits the capacity of pathogens to exert their impact (Xia et al., 2015). Thus, the present experiment was conducted in two settings as previous described (Xia et al., 2015). First, IPEC-J2 cells were treated with biogenic MccJ25 for 1 h, then cells were washed off before bacterial addition or in culture medium during infection. We observed that washed out or consistent organism biogenic MccJ25, at a concentration of 2 μg/ml, can significantly reduce the number of ETEC K88. Consistent with our study, a report from Brown et al. (2018) has been showed that salivary peptide can block adhesion of ETEC to intestinal epithelial cells.

Intracellular or extracellular pressure can lead to impaired mucosal barrier function and increased release of LDH from cell culture media (Jiao et al., 2015; Ma et al., 2017). Another typical and convenient indicator of epithelial integrity is TEER, which is an indicator of intestinal epithelial permeability. High TEER means low cell-cell epithelial permeability (Jiao et al., 2015). To address this hypothesis, the LDH and TEER were determined. We observed that pretreatment with biogenic MccJ25 can significantly reduce ETEC K88 adhesion in cells and attenuate ETEC K88-induced increases in LDH release and decreases in the TEER in epithelial cells even without the contact with the ETEC K88. These studies suggest that biogenic MccJ25 Physical barriers can be formed between pathogens and intestinal cells or induce intestinal epithelial cell characteristics that may be opposed to infection.

Based on our analysis above, to further elucidate how biogenic MccJ25 mediates its impact on barrier function, we determined the expression of TJPs in IPEC-J2 cells. Notably, several AMPS, including porcin β-defensin 2 (pBD2) and Cathelicidin-WA (CWA) appear to affect the expression of ZO, occludins and claudin *in vivo* and *in vitro* (Han et al., 2015; Zhang et al., 2015). Consist with these findings, in our study, ETEC K88 challenge reduced mRNA and protein abundance of claudin-1 and occludin, but these reductions were prevented by pre-treatment with biogenic MccJ25. Interestingly, compared with control group, biogenic MccJ25 treatment group did not significantly affect the protein expression of ZO-1, this is not surprising, because this discrepancy may be a possible variation in the origin of the peptides (natural or synthetic) or mode of action of peptides. Additionally, immunofluorescent staining of IPEC-J2 cells for claudin and occludin indicated a distinct organization of these intercellular tight junction proteins, considering the lack of well-developed TJPs in IPEC-J2 cell monolayers. Therefore, these findings indicated that biogenic MccJ25 can protects the intestinal epithelial integrity by direct killing the pathogens and forms a physical barrier (increased in the assembly of claudin-1 and occludin in the tight bound complexes or TEER) between pathogens and intestinal cells and/or induce intestinal cell characteristics that are resistant to infection. This confirms previous studies that reported that AMP can increase tight junction integrity and enhance intestinal barrier function and permeability to fight pathogen infections (Xia et al., 2015; Yi et al., 2016).

Meanwhile, epithelial barrier damage is usually associated with immune-mediated disorders (Ling et al., 2016; Yi et al., 2017; Fuente-Nunez et al., 2018). The ability of the peptides to skew host responses to favor cellular recruitment. At the same time, controlling excessively harmful inflammation makes these peptides ideal candidates for treating acute infections and conditions of the intestinal tract (Oshitani et al., 2005; Wang et al., 2017). Various studies reported that humans cathelicidin LL-37 and AMPs sublancin and biogenic MccJ25 are known to exert several immunomodulation activities in cell lines and in animals (Zasloff, 2002; Nijnik and Hancock, 2009; Brown et al., 2011; Wang et al., 2017; Yu et al., 2017). In the present study, pretreatment with the biogenic MccJ25 reduced the ETEC K88-induced the secretion and gene expression of pro-inflammatory cytokines such as IL-6, IL-8, and TNF-α. Previous studies have also demonstrated that ETEC induces the production of pro-inflammatory cytokines in epithelial cells that may alter attachment sites and cytoskeletal reorganization, regulate cell barrier function, and create an inflammatory environment around the epithelial barrier (Wu et al., 2016).

Regulation of pro-inflammatory cytokines expression involves MAPK and NF- κB signaling activation (Ling et al., 2016; Wu et al., 2016). Various studies have established correlations between TNF-α, IL-6, or IL-8 and intestinal permeability, and speculated that NF-κB and MAPK signaling molecules play a role in the expression of pro-inflammatory cytokines (Zhang et al., 2005; Suzuki et al., 2011; Al-Sadi et al., 2014), for instance, activation of the p38 MAPK and NF-κB pathways and promotes TNF-α, IL-6 and IL-8 production and gene expression *in vitro* and *in vivo* (Wang et al., 2008; Ulluwishewa et al., 2011). In this study, our results clearly show that biogenic MccJ25 may significantly inhibit the expression of ETEC-triggered inflammatory cytokines by down-regulating MAPK and NF-κB pathways. This explanation is consistent with the reduction of pathogen-induced IL-8, IL-6 AMP, and TNF-α production in intestinal epithelial cells by affecting NF-κB pathway in cells (Nijnik and Hancock, 2009; Wang et al., 2017). Additionally, some previous reports have shown the link between MAPK activation and barrier dysfunction, which may be mediated/linked by pro-inflammatory cytokines. TJ adjustments (repair, assembly, and disassembly) were proposed for different physiological and pathological conditions (Ulluwishewa et al., 2011; Ling et al., 2016). Therefore, further investigation of other signaling pathways is necessary to elucidate the possible other effects of biogenic MccJ25 on TJ regulation.

In summary, our findings suggest that biogenic MccJ25 exhibited strong antimicrobial activity to ETEC-K88 without toxicity toward IPEC-J2 cells. Additionally, the present study also demonstrates that pretreatment of intestinal cells with biogenic MccJ25 prevented ETEC K88-induced intestinal damage and reduced inflammation. Despite the many limitations of this *in vitro* method, it is important to use this data to inform development of more detailed animal models (e.g., mice or pigs) and subsequent studies in humans. This may provide biogenic MccJ25 with great potential for the treatment of infectious diseases caused by pathogens and contribute to the development of potentially effective antibiotic-independent methods to control the use of ETEC K88 in human and other animal depots.

## Author contributions

XZ and SQ designed the experiments. HY, XD, LS, HL, and NL performed the experiments. HY, XZ, and SH analyzed the data. HY wrote the paper, which was edited by CL, LJ, XZ, and SQ. YW, GW, SC, and MC contributed reagents, materials, analysis tools.

### Conflict of interest statement

The authors declare that the research was conducted in the absence of any commercial or financial relationships that could be construed as a potential conflict of interest.

## Abbreviations

MccJ25: Microcin J25
IPEC-J2: intestinal porcine epithelial cells J2
ETEC K88: Enterotoxigenic *Escherichia coli* K88
LDH: lactate dehydrogenase
TJPs: tight junction proteins
TNF-α: tumor necrosis factor alpha
ZO: zonula occludens
CFU: colony forming units
DMEM-F12, HRP: horseradish peroxidase
ITS: Insulin-transferrin-selenium.

## References

[B1] Ahmed I. Roy B. C. Khan S. A. Septer S. Umar S. (2016). Microbiome, metabolome and inflammatory bowel disease. Microorganisms 4:20. 10.3390/microorganisms4020020 27681914 PMC5029486

[B2] Al-Sadi R. Ye D. Boivin M. Guo S. Hashimi M. Ereifej L.. (2014). Interleukin-6 modulation of intestinal epithelial tight junction permeability is mediated by JNK pathway activation of claudin-2 gene. PLoS ONE 9:e85345. 10.1371/journal.pone.0085345 24662742 PMC3963839

[B3] Black R. E. (1990). Epidemiology of travelers' diarrhea and relative importance of various pathogens. Rev. Infect. Dis. 1, S73–S79. 2406860 10.1093/clinids/12.supplement_1.s73

[B4] Blond A. Péduzzi J. Goulard C. Chiuchiolo M. J. Barthelemy M. Prigent Y.. (1999). The cyclic structure of microcin J25, a 21-residue peptide antibiotic from *Escherichia coli*. Eur. J. Biochem. 259, 747–755. 10.1046/j.1432-1327.1999.00085.x 10092860

[B5] Boucher H. W. Talbot G. H. Bradley J. S. Edwards J. E. Gilbert D. Rice L. B.. (2009). Bad bugs, no drugs: no ESKAPE! An update from the Infectious Diseases Society of America. Clin. Infect. Dis. 48, 1–12. 10.1086/595011 19035777

[B6] Brown J. W. Badahdah A. Iticovici M. Vickers T. J. Alvarado D. M. Helmerhorst E. J.. (2018). A role for Salivary peptides in the innate defense against enterotoxigenic *Escherichia coli*. J. Infect. Dis. 217, 1435–1441. 10.1093/infdis/jiy032 29528423 PMC5894089

[B7] Brown K. L. Poon G. F. T. Birkenhead D. Pena O. M. Falsafi R. Dahlgren C.. (2011). Host defense peptide LL-37 selectively reduces proinflammatory macrophage responses. J. Immunol. 186, 5497–5505. 10.4049/jimmunol.1002508 21441450

[B8] Cao J. C. Fuente-Nunez C. D. L. Ou R. W. Torossian T. M. D. Santosh G. Pande S. G.. (2018). Yeast-based synthetic biology platform for antimicrobial peptide production. ACS Synth. Biol. 7, 896–902. 10.1021/acssynbio.7b00396 29366323

[B9] Chen J. Li Y. Tian Y. Huang C. Li D. F. Zhong Q.. (2015). Interaction between microbes and host intestinal health: modulation by dietary nutrients and gut-brain-endocrine- immune axis. Curr. Protein. Pept. Sci. 16, 592–603. 10.2174/1389203716666150630135720 26122779

[B10] Chen X. Zhu F. M. Cao Y. H. Qiao S. Y. (2009). Novel expression vector for secretion of Ceropin AD in *Bacillus subtilis* with enhanced antimicrobial activity. Antimicrob. Agents Chemother. 53, 3683–3689. 10.1128/AAC.00251-09 19546372 PMC2737859

[B11] Donato K. A. Gareau M. G. Wang Y. J. J. Sherman P. M. (2010). *Lactobacillus rhamnosus* GG attenuates interferon-gamma and tumour necrosis factor-alpha-induced barrier dysfunction and pro-inflammatory signaling. Microbiology 156, 3288–3297. 10.1099/mic.0.040139-0 20656777

[B12] Farhadi A. Banan A. Fields J. Keshavarzian A. (2003). Intestinalbarrier:an interface between health and disease. J. Gastroenterol. Hepatol. 18, 479–497. 10.1046/j.1440-1746.2003.03032.x 12702039

[B13] Fuente-Nunez C. D. L. Meneguetti B. T. Franco O. L. Lu T. K. (2018). Neuromicrobiology: how microbes influence the brain. ACS Chem. Neurosci. 9, 141–150. 10.1021/acschemneuro.7b00373 29220570

[B14] Han F. F. Zhang H. W. Xia X. Xiong H. T. Song D. G. Zong X.. (2015). Porcine β-defensin 2 attenuates inflammation and mucosal lesions in dextran sodium sulfate-induced colitis. J. Immunol. 194, 1882–1893. 10.4049/jimmunol.1402300 25601921

[B15] Herbel V. Schäfer H. Wink M. (2015). Recombinant production of Snakin-2 (an antimicrobial peptide from tomato) in *E. coli* and analysis of its bioactivity. Molecules 20, 14889–14901. 10.3390/molecules200814889 26287145 PMC6332222

[B16] Hooper L. V. Gordon J. I. (2001). Commensal host-bacterial relationships in the gut. Science 292, 1115–1118. 10.1126/science.1058709 11352068

[B17] Jiang Q. Zhang H. Xie Y. Wang Y. (2016). Recombinant expression of porcine lactoferrin peptide LF-6 with intein technology and its immunomodulatory function in ETEC K88-infected mice. Int. Immunopharmac. 39, 181–191. 10.1016/j.intimp.2016.07.029 27487204

[B18] Jiao N. Wu Z. L. Ji Y. Wang B. Dai Z. L. Wu G. Y. (2015). L-Glutamate enhances barrier and antioxidative functions in intestinal porcine epithelia cells. J. Nutr. 145, 2258–2264. 10.3945/jn.115.217661 26338884

[B19] Johnson A. M. Kaushik R. S. Hardwidge P. R. (2010). Disruption of transepithelial resistance by enterotoxigenic *Escherichia coli*. Vet. Microbiol. 141, 115–119. 10.1016/j.vetmic.2009.08.020 19733985

[B20] Landy J. Ronde E. English N. Clark S. K. Hart A. L. Knight S. C.. (2016). Tight junctions in inflammatory bowel diseases and inflammatory bowel disease associated colorectal cancer. World. J. Gastroenterol. 22, 3117–3126. 10.3748/wjg.v22.i11.3117 27003989 PMC4789987

[B21] Ling K. H. Wan M. L. El-Nezami H. Wang M. (2016). Protective capacity of resveratrol, a natural polyphenolic compound, against deoxynivalenol-induced intestinal barrier dysfunction and bacterial translocation. Chem. Res. Toxicol. 29, 823–833. 10.1021/acs.chemrestox.6b00001 27058607

[B22] Liu H. Hou C. Wang G. Jia H. Yu H. Zeng X.. (2017). *Lactobacillus reuteri* I5007 modulates intestinal host defense peptide expression in the model of IPEC-J2 cells and neonatal piglets. Nutrients 9:E559. 10.3390/nu9060559 28561758 PMC5490538

[B23] Lu R. Y. Yang W. X. Hu Y. J. (2014). The role of epithelial tight junctions involved in pathogen infections. Mol. Biol. Rep. 41, 6591–6610. 10.1007/s11033-014-3543-5 24965148

[B24] Ma Z. X. Garrido-Maestu A. Lee J. Chon J. Jeong D. Yue Y. R.. (2017). Comprehensive *in vitro* and *in vivo* risk assessments of chitosan microparticles using human epithelial cells and *Caenorhabditis elegans*. J. Hazard. Mater. 341, 248–256. 10.1016/j.jhazmat.2017.07.071 28797941

[B25] Ma Z. X. Kim D. Adesogan A. T. Ko S. Galvao K. N. A. Jeong K. C. (2016). Chitosan microparticles expert broad-spectrum antimicrobial activity against antibiltic-resistant microorganisms without increasing resistance. ACS Appl. Mater. Interfaces 8, 10700–10709. 10.1021/acsami.6b00894 27057922

[B26] Nijnik A. Hancock R. E. W. (2009). The roles of cathelicidin LL-37 in immune defences and novel clinical applications. Curr. Opin. Hematol. 16, 41–47. 10.1097/MOH.0b013e32831ac517 19068548

[B27] Osek J. (2000). Clonal analysis of *Escherichia coli* strains isolated from pigs with post- weaning diarrhea by pulsed-field gel electrophoresis. FEMS Microbiol. Lett. 186, 327–331. 10.1111/j.1574-6968.2000.tb09125.x 10802192

[B28] Oshitani N. Watanabe K. Nakamura S. Fujiwara Y. Higuchi K. Arakawa T. (2005). Dislocation of tight junction proteins without F-actin disruption in inactive Crohn's disease. Int. J. Mol. Med. 15, 407–410. 10.3892/ijmm.15.3.407 15702229

[B29] Pan S. J. (2012). Biogenic and Engineering of Lasso Peptides. Ph.D. dissertation. New York, NY: University of Princeton.

[B30] Park S. H. Choi H. J. Yang H. Do K. H. Kim J. Moon Y. (2010). Repression of peroxisome proliferator-activated receptor gamma by mucosal ribotoxic insult-activated CCAAT/ enhancer-binding protein homologous protein. J. Immunol. 185, 5522–5530. 10.4049/jimmunol.1001315 20889551

[B31] Qin H. L. Zhang Z. W. Hang X. M. Jiang Y. Q. (2009). *L. plantarum* prevents enteroinvasive *Escherichia coli-*induced tight junction proteins changes in intestinal epithelial cells. BMC Microbiol. 9:63. 10.1186/1471-2180-9-63 19331693 PMC2674056

[B32] Rabanal F. Grau-Campistany A. Vila-Farres X. Gonzalez-Linares J. Borras M. Vila J.. (2015). A bioinspired peptide scaffold with high antibiotic activity and low *in vivo* toxicity. Sci. Rep. 5:10558. 10.1038/srep10558 26024044 PMC4603705

[B33] Sable S. Pons A. M. Gendron-Gaillard S. Cottenceau G. (2000). Antibacterial activity evaluation of microcin J25 against diarrheagenic *Escherichia coli*. Appl. Environ. Microb. 66, 4595–4597. 10.1128/AEM.66.10.4595-4597.2000 11010926 PMC92352

[B34] Salomón R. A. Farías R. N. (1992). Microcin 25, a novel antimicrobial peptide produced by *Escherichia coli*. J. Bacteriol. 174, 7428–7435. 10.1128/jb.174.22.7428-7435.1992 1429464 PMC207439

[B35] Sassone-Corsi M. Nuccio S. P. Liu H. Hernandez D. Vu C. T. Takahashi A. A.. (2016). Microcins mediate competition among Enterobacteriaceae in the inflamed gut. Nature. 540, 280–283. 10.1038/nature20557 27798599 PMC5145735

[B36] Schmidt L. D. Kohrt L. J. Brown D. R. (2008). Comparison of growth phase on Salmonella enterica serovar Typhimurium invasion in an epithelial cell line (IPEC J2) and mucosal explants from porcine small intestine. Comp. Immunol. Microbiol. Infect. Dis. 31, 63–69. 10.1016/j.cimid.2007.04.003 17544508 PMC10656783

[B37] Skjolaas K. A. Burkey T. E. Dritz S. S. Minton J. E. (2006). Effects of Salmonella enterica serovars Typhimurium (ST) and Choleraesuis (SC) on chemokine and cytokine expression in swine ileum and jejunal epithelial cells. Vet. Immunol. Immunopathol. 111, 199–209. 10.1016/j.vetimm.2006.01.002 16473412

[B38] Suzuki T. Yoshinaga N. Tanabe S. (2011). Interleukin-6 (IL-6) regulates claudin-2 expression and tight junction permeabilityin intestinal epithelium. J. Biol. Chem. 286, 31263–31271. 10.1074/jbc.M111.238147 21771795 PMC3173073

[B39] Ulluwishewa D. Anderson R. C. McNabb W. C. Moughan P. J. Wells J. M. Roy N. C. (2011). Regulation of tight junction permeability by intestinal bacteria and dietary components. J. Nutr. 141, 769–776. 10.3945/jn.110.135657 21430248

[B40] Wang Q. Guo X. L. Wells-Byrum D. Noel G. Pritts T. A. Ogle C. K. (2008). Cytokine-induced epithelial permeability changes are regulated by the activation of the p38 mitogen-activated protein kinase pathway in cultured Caco-2 cells. Shock 29, 531–537. 10.1097/SHK.0b013e318150737f 17724435

[B41] Wang Q. W. Zeng X. F. Wang S. Hou C. L. Yang F. J. Ma X.. (2014). The bacteriocin sublancin attenuates intestinal injury in young mice infected with *Staphylococcus aureus*. Anat. Rec. 297, 1454–1461. 10.1002/ar.22941 24809978

[B42] Wang S. Wang Q. Zeng X. Ye Q. Huang S. Yu H.. (2017). Use of the antimicrobial peptide sublancin with combined antibacterial and immunomodulatory activities to protect against methicillin-resistant *Staphylococcus aureus* infection in mice. J. Agric. Food. Chem. 65, 8595–8605. 10.1021/acs.jafc.7b02592 28906115

[B43] Wu S. D. Zhang F. R. Huang Z. M. Liu H. Xie C. Y. Zhang J.. (2012). Effects of the antimicrobial peptide cecropin AD on performance and intestinal health in weaned pigs challenged with *Escherichia coli*. Peptides 35, 225–230. 10.1016/j.peptides.2012.03.030 22490448

[B44] Wu Y. Zhu C. Chen Z. Chen Z. J. Zhang W. N. Ma X. Y.. (2016). Protective effects of *Lactobacillus plantarum* on epithelial barrier disruption caused by enterotoxigenic *Escherichia coli* in intestinal porcine epithelial cells. Vet. Immunol. Immunopathol. 172, 55–63. 10.1016/j.vetimm.2016.03.005 27032504

[B45] Xia X. Zhang L. Wang Y. Z. (2015). The antimicrobial peptide cathelicidin-BF could be a potential therapeutic for *Salmonella typhimurium* infection. Microbiol Res. 171, 45–51. 10.1016/j.micres.2014.12.009 25644952

[B46] Xiao J. Zhang H. Niu L. Wang X. (2011). Efficient screening of a novel peptide from *Jatropha curcas* by cell membrane affinitychromatography. J. Agr. Food Chem. 59, 1145–1151. 10.1021/jf103876b 21268582

[B47] Yi H. B. Hu W. Y. Chen S. Lu Z. Q. Wang Y. Z. (2017). Cathelicidin-WA improves intestinal epithelial barrier function and enhances host defence against enterohemorrhhagic *Escherichia coli* O157:H7 infection. J. Immunol. 198, 1696–1705. 10.4049/jimmunol.1601221 28062699

[B48] Yi H. Zhang L. Gan Z. Xiong H. Yu C. Du H.. (2016). High therapeutic efficacy of Cathelicidin-WA against postweaning diarrhea via inhibiting inflammation and enhancing epithelial barrier in the intestine. Sci. Rep. 6:25679. 10.1038/srep25679 27181680 PMC4867772

[B49] Yu H. T. Ding X. L. Li N. Zhang X. Y. Zeng X. F. Wang S.. (2017). Dietary supplemented antimicrobial peptide Microcin J25 improves the growth performance, apparent total tract digestibility, fecal microbiota, and intestinal barrier function of weaned pigs. J. Anim. Sci. 95, 5064–5076. 10.2527/jas2017.1494 29293710 PMC6292272

[B50] Yuhan R. Koutsouris A. Savkovic S. D. Hecht G. (1997). Enteropathogenic *Escherichia coli*-induced myosin light chain phosphorylation alters intestinal epithelial permeability. Gastroenterology 113, 1873–1882. 10.1016/S0016-5085(97)70006-4 9394726

[B51] Zasloff M. (2002). Antimicrobial peptides of multicellular organisms. Nature 415, 389–395. 10.1038/415389a 11807545

[B52] Zhang H. Xia X. Han F. Jiang Q. Rong Y. Song D.. (2015). Cathelicidin-BF, a novel antimicrobial peptide from *Bungarus fasciatus*, attenuates disease in a dextran sulfate sodium model of colitis. Mol. Pharmaceutics. 12, 1648–1661. 10.1021/acs.molpharmaceut.5b00069 25807257

[B53] Zhang L. Y. Li N. Caicedo R. Neu J. (2005). Alive and dead *Lactobacillus rhamnosus* GG decrease tumor necrosis factor-α induced interleukin-8 production in caco-2 cells. *J*. Nutr. 135, 1752–1756. 10.1093/jn/135.7.1752 15987860

[B54] Zhang M. Sun K. J. Wu Y. J. Yang Y. Tso P. Wu Z. L. (2017). Interactions between intestinal microbiota and host immune response in inflammatory bowl disease. Front. Immunol. 8:942. 10.3389/fimmu.2017.00942 28855901 PMC5558048

[B55] Zong X. Hu W. Y. Song D. G. Li Z. Du H. H. Lu Z. Q.. (2016). Porcine lactoferrin-derived peptide LFP-20 protects intestinal barrier by maintaining tight junction complex and modulating inflammatory. Biochem. Pharmacol. 104, 73–82. 10.1016/j.bcp.2016.01.009 26776304

