# Supplementary material for: Protective Ability of Biogenic Antimicrobial Peptide Microcin J25 Against Enterotoxigenic Escherichia Coli-Induced Intestinal Epithelial Dysfunction and Inflammatory Responses IPEC-J2 Cells
Source: Front Cell Infect Microbiol. 2018 Jul 13;8:242. doi: 10.3389/fcimb.2018.00242 (PMC6053529; doi:10.3389/fcimb.2018.00242)
Supplement: Supplementary file 1 [file Presentation_1.PDF]

Supporting Information

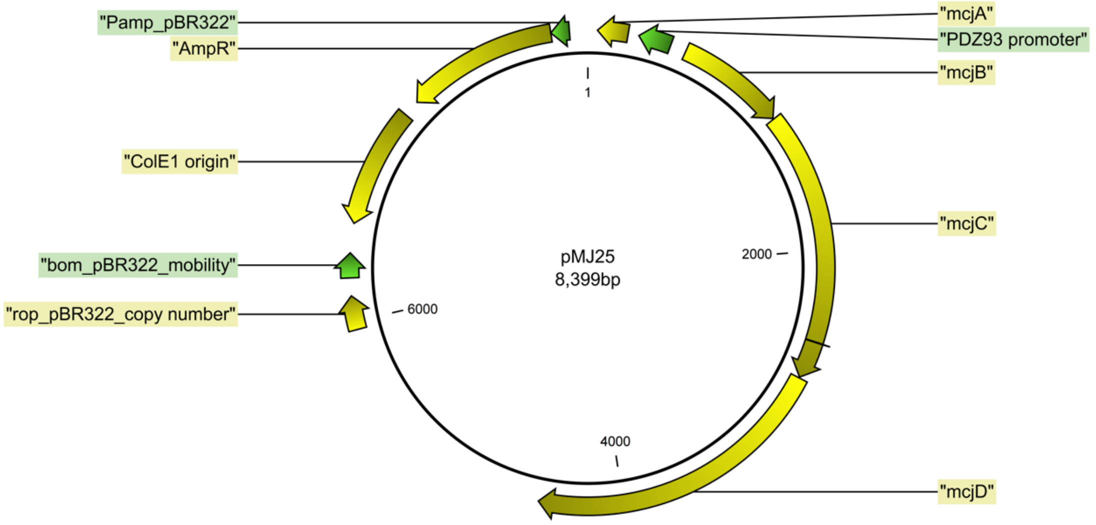

Figure S1. Schematic representation of the pMJ25 expression vector and detailed information about biogenic antimicrobial peptide MccJ25.

PRODUCTION and PURIFICATION of BIOGENIC AMP MccJ25

The recombinant bacteria were cultured in sucrose-complex medium at 37°C with 100 μg/L ampicillin in 10L fermenter for 22 h. The optimum medium: sucrose 5.2 g/L, peptone 19.1 g/L, yeast extract powder 26 g/L, potassium dihydrogen phosphate, 1 g/L, manganese sulfate·$4H_2O$ 0.1 g/L, magnesium sulfate·$7H_2O$ 0.5 g/L. Biogenic AMP MccJ25 purified as follows:

Step 1: After incubation, cell supernatant was harvested by centrifuge (12000rpm for 10 min at 4°C) and filtered twice using an Amicon ultrafiltration device (Millipore, Billerica, MA).

Step 2: Ammonium sulfate precipitation treatment. The supernatant was treated with ammonium sulfate. The mixtures were separated by centrifuge (1000 rpm/min for 10 min at 4°C) and the residue was dissolved in water.

Step 3: MccJ25 was purified by His-tag affinity purification and desalting (GE AKTA AVANT 150) using a column Hydrophobic Interaction Chromatography (Butyl Sepharose) with 1 M/L Nacl and ultrapure water as Buffer A and Buffer B, respectively.

the column 2CV (column volume) was washed with buffer A. MccJ25 is eluted with linear gradient of buffer B increasing from 0% to 100%. When increasing to 10%, the target peak is collected, frozen dryness and the gel (sephadex-G10, column length 20cm, column diameter 2.6cm;) Then, ethanol isometric elution is used to remove salt.
