# Supplementary material for: Protective Ability of Biogenic Antimicrobial Peptide Microcin J25 Against Enterotoxigenic Escherichia Coli-Induced Intestinal Epithelial Dysfunction and Inflammatory Responses IPEC-J2 Cells
Source: Front Cell Infect Microbiol. 2018 Jul 13;8:242. doi: 10.3389/fcimb.2018.00242 (PMC6053529; doi:10.3389/fcimb.2018.00242)
Supplement: Supplementary file 2 [file Table_1.DOCX]

**TABLE 1 |** Sequences of oligonucleotide primers for gene expression using real-time PCR.

| Gene | Primer Sequence | Product Size | GeneBank Accession No. |
| --- | --- | --- | --- |
| *β*-actin | F:5'-TGCGGGACATCAAGGAGAAG-3'  R: 5'-AGTTGAAGGTGGTCTCGTGG-3' | 217 | DQ845171 |
| TNF-α | F:5'-GCATGGTGGTGGTTGTTTCTGACGAT-3'  R: 5'-GCTTCTGTTGGACACCTGGAGACA-3' | 99 | NM_010851.2 |
| IL-6 | F: 5'-GGCTGCTTCTGGTGATGGCTA-3'  R: 5'-TTGCCTCAGGGTCTGGATCAGT-3' | 416 | NM_001252429.1 |
| IL-8 | F: 5'-TCTCGGTGTAGAGCAAGG-3'  R: 5'-TTCCCAAAGTGCTGGTATT-3' | 146 | NM_011339.2 |
| ZO-1 | F: 5'-AAGGATGTTTACCGTCGCATT-3'  R: 5'-ATTGGACACTGGCTAACTGCT-3' | 253 | XM_003353439.2 |
| claudin-1 | F: 5'-GCTGGGTTTCATCCTGGCTTCT-3'  R: 5'-CCTGAGCGGTCACGATGTTGTC-3' | 110 | NM_016674.4 |
| occludin | F: 5'-GTGGTAACTTGGAGGCGTCTTC-3'  R: 5'-CCGTCGTGTAGTCTGTCTCGTA--3' | 102 | NM_001163647.2 |
